# HbA1c levels and circulating inflammatory proteins at onset of type 1 diabetes in children and adolescents

**DOI:** 10.1007/s40200-022-01075-3

**Published:** 2022-07-07

**Authors:** Jonatan Dereke, Charlotta Nilsson

**Affiliations:** 1 Department of Clinical Sciences, Diabetes Research Laboratory, Lund University, Lund, Sweden; 2 Department of Pediatrics, Department of Clinical Sciences, Helsingborg Hospital, Lund University, S- 251 87 Helsingborg, Sweden

**Keywords:** Type 1 diabetes, HbA1c, Children, Inflammatory, Complications

## Abstract

**Purpose:**

Type 1 diabetes is an autoimmune disease that often develops during childhood. Complications such as retinopathy often occur during the course of the disease. Studies to identify possible predictors of complications in type 1 diabetes are needed; in particular markers able to identify risk of complications long before they occur. The first aim of this study was to investigate plasma levels of sCD163, sST2 and Gal-3 at diagnosis of type 1 diabetes in children and adolescents. The second aim was to study their correlation to HbA1c in this study cohort.

**Methods:**

Patients (n = 242, 0–18 years) with type 1 diabetes, at Helsingborg’s Hospital were included in this study and circulating levels of sCD163, sST2 and Gal-3 were investigated in plasma using commercially available DuoSet ELISA and supplementary ancillary kit.

**Results:**

Circulating sCD163 was significantly higher at diagnosis compared to after diagnosis (666 ± 318ng/ml and 505 ± 223ng/ml respectively; p < 0.001). Also sST2 was significantly higher (18.2 [12.7–25.6] ng/ml respectively 9.1 [6.3–13.5] ng/ml (p < 0.001), but Gal-3 levels did not differ from onset of diabetes to after diagnosis. HbA1c was shown to correlate to sCD163 (r_s_=0.36; p < 0.001), sST2 (r_s_=0.22; p = 0.016) and Gal-3 (r_s_=0.2; p = 0.020) in patients with a diabetes duration < 5 years.

**Conclusions:**

sCD163 levels increased in patients with recent-onset type 1 diabetes and the levels increased with higher HbA1c. Patients included in this study will be followed annually until the eventual development of diabetic complications, while continuously studying circulating levels of inflammatory proteins such as sCD163.

## Introduction

Type 1 diabetes mellitus is an autoimmune disease that affects all ages but is more common at diagnosis among children and adolescents [1]. Sweden has one of the highest incidences of type 1 diabetes around the world and the global increase of type 1 diabetes during childhood is 3% every year [2]. It seems that the highest increase is among children under the age of five [3] and when reaching adulthood they already have had diabetes for a long time. Complications often occur during the course of the disease. The most common complications are retinopathy, nephropathy, peripheral neuropathy and autonomic neuropathy [1]. To avoid or delay complications from diabetes the most important thing is strict glycaemic control [4]. Glycosylated haemoglobin (HbA1c) reflects the average blood glucose level over the recent 8–12 weeks and is therefore useful when predicting the long-term risk of complications from type 1 diabetes [5]. In Sweden, HbA1c levels ≤ 48 mmol/mol without the presence of severe hypoglycaemia or frequent mild hypoglycaemia is the general target for optimal glycaemic control in children and adolescents. These days with continuous glucose monitoring (CGM), Time in Range (TIR), defined as blood glucose between 3.9 and 10.0 mmol/L, also is used as clinical target [6].

Besides blood glucose levels one of the most common risk factors for developing complications from type 1 diabetes is diabetes duration and signs of retinopathy can be observed after only 10 years with the disease [7, 8]. Among other risk factors are high blood pressure and cholesterol levels [1]. Recently, studies have suggested soluble biomarkers which may identify the increased risk of developing diabetes-related complications in adults [9] and young patients with type 1 diabetes [10]. These studies have found several markers correlating with risk for vascular complications in type 1 diabetes. Additional studies to identify possible predictors of complications in type 1 diabetes are needed; in particular markers able to identify the risk of complications long before they occur.

CD163 is a glycosylated membrane-bound protein involved in haemoglobin-haptoglobin binding and expressed on monocytes and macrophages active in adipose tissue [11]. Enzymatic cleavage leading to ectodomain shedding results in soluble CD163 (sCD163) attenuating its haemoglobin-haptoglobin binding ability [12]. sCD163 is released concomitantly with TNF-α as a result of the activation of TACE (TNF-α converting enzyme) and has been suggested to be a more stable marker of TNF-α release. Serum levels of sCD163 have been reported to be an independent predictor of future type 2 diabetes development and increased in men with type 1 diabetes [13, 14]. Circulating levels of sCD163 in healthy adults have been measured to 0.7-3.9 mg/l [15]. We have previously reported plasma levels of sCD163 to be linked to galectin-3 (Gal-3) and diabetic retinopathy in adults with type 1 diabetes [16].

Soluble ST2 (sST2) is the secreted splice variant of the IL-33 receptor, ST2, which is expressed on Th2-cells, mast cells and granulocytes among others. IL-33 via ST2 and IL-1R accessory protein signalling induces NFκB and MAPK activation [17]. sST2 is thought to act as a decoy receptor for IL-33 and by mitigating IL-33/ST2 signalling, has an anti-inflammatory effect [18]. sST2 has been reported to be increased in patients with type 2 diabetes[19]. Our previous research has shown that plasma sST2 in patients at the onset of diabetes is also independently associated with the later development of diabetic nephropathy [20].

Gal-3 is a soluble β-galactoside binding lectin expressed in numerous cell types and implicated in a range of biological processes including immune activation, cell proliferation, fibrosis and host defence [21, 22]. Elevated Gal-3 plasma levels have been reported in a meta-analysis including over 30,000 participants to be associated with a higher risk of all-cause mortality, cardiovascular mortality and heart failure [23].

## Aim

The first aim of this study was to investigate plasma levels of sCD163, sST2 and Gal-3 at diagnosis of type 1 diabetes in children and adolescents. The second aim was to study soluble proteins correlation to HbA1c in this study cohort.

.

## Methods

### Patients

Children and adolescents (up to the age of 18) diagnosed with type 1 diabetes at the Paediatric Department at Helsingborg Hospital in Sweden were included in this study. All children received oral and written information about the study. For children up to 15 years of age their parents signed the written consent to participate in the study and from the age of 15 the adolescent, as well as their parents, signed the written consent. Blood samples were collected from the participants of this study between 2016 and 2020. For those with clinical onset and diagnosis of type 1 diabetes (n = 74) and who were asked about the study, the participation rate was 100%. Two patients were missed and not asked about the study. For children at the Department of Paediatrics with pre-existing type 1 diabetes when the study started (n = 168), their blood samples were collected once a year at the same time as their regular annual blood tests were taken and among these children and adolescents, two patients choose not to participate.

The children´s age, weight, height and HbA1c were noted when the blood sample was collected.

The study was approved by the Regional Ethical Review Board in Lund, Sweden, Dnr 2006/599, 2013/693 and 2014/822 and the study was in accordance with the declaration of Helsinki.

### Laboratory methods

Circulating levels of sCD163, sST2 and Gal-3 were investigated in plasma using commercially available DuoSet ELISA kits (#DY1607, #DY523B-05 and #DY1154) and supplementary ancillary kit (#DY008; R&D Systems, Minneapolis, MN, USA). Participant plasma samples were diluted 1:200, 1:20 or 1:2 in PBS with 1% BSA for sCD163, sST2 and Gal-3 respectively. The diluted samples were run in duplicates and subgroups were alternated on each ELISA plate to minimize bias caused by intra- and inter-variations. The ELISA analyses were run in accordance with the manufacturer’s instructions. Absorbance was measured at 450 nm using a FLOUstar Optima plate reader (BMG Labtech Gmbh, Ortenburg, Germany). Sample concentrations were predicted by plotting a 4-parameter fit using a 7-point standard curve. The intra- and inter-coefficient of variation reached 1.9% and 5.1%, 3.9%, and 6.0%, and 3.1% and 12.4% for sCD163, sST2 and Gal-3 respectively.

HbA1c values at type 1 diabetes diagnosis were analyzed in Capillarys TERA Hemoglobin A1c Kits [24] and thereafter in Alere Afinion AS100 analyzer [25]. For HbA1c IFCC-units mmol/mol were used.

### Statistical analyses

Normality of continuous data was estimated prior to statistical analysis by observing the skewness and shape of the distribution. Data deemed normal was presented as mean ± SD, while non-normal data was presented as median followed by interquartile range in brackets. Differences in continuous variables were investigated between groups using ANOVA or the Kruskal-Wallis *H*-test depending on normality. Tukey-Kramer or Conover post-hoc tests was applied to statistically significant ANOVA and Kruskal-Wallis *H*-tests for pairwise comparison of subgroups. Paired samples t-test or the Wilcoxon test for paired samples was used to estimate differences in patients at baseline and follow-up. Correlations in continuous variables were investigated with regression analyses and non-normal variables were log-transformed prior to analysis. P-values < 0.05 were considered statistically significant. Multiple regression analysis was used to investigate confounding effects on correlations. All statistical analyses were performed using MedCalc Statistical Software version 19.2.6 (MedCalc Software Ltd, Ostend, Belgium; https://www.medcalc.org; 2020).

## Results

The diabetes duration for patients with pre-existing type 1 diabetes when the study started, was 1–16 years. Measuring analyses in plasma using commercial ELISA showed that there are significantly elevated levels of sCD163 in patients with a diabetes duration < 5 years (508 [378–699] ng/ml) compared to patients with a longer diabetes duration; 5–10 years (414 [334–510] ng/ml) and > 10 years (394 [344–576] ng/ml; p = 0.001; Table 1). No significant difference in sST2 (p = 0.25) nor Gal-3 (p = 0.87) levels could be observed with regard to diabetes duration. Circulating sCD163 was inversely correlated to patient age (p < 0.001) and sST2 positively correlated to age (p < 0.001). Gal-3 did not correlate with patient age. Multiple regression showed that sCD163 (p < 0.001) and sST2 (p = 0.005) remained correlated to HbA1c when adjusting for patient age.

**Table 1:** **Demographic information and biochemical data for the study participants**. ANOVA or Kruskal-Wallis *H*-test has been used to compare variables between groups. A statistically significant increase in sCD163 levels could be observed in patients with a diabetes duration < 5 years

	< 5 years (n = 137)	5–10 years (n = 72)	> 10 years (n = 33)	P-value
Age (years)	11.0 ± 4.5	13.5 ± 2.8	15.4 ± 2.3	< 0.001
BMI (kg/m^2^)	19.2 ± 4.6	20.9 ± 4.3	22.1 ± 4.2	< 0.001
Sex (m/f)	72/65 (53%)	45/27 (63%)	18/15 (55%)	0.47
Diabetes duration (years)	0.0 [0.0–3.0]	7.0 [6.0–8.0]	11.0 [10.8–13.0]	N/A
HbA1c (mmol/mol)	65.0 [50.1–88.5]	55.0 [50.0–65.0]	61.5 [56.5–67.0]	0.005
sCD163 (ng/ml)	508 [378–699]	414 [334–510]	394 [344–576]	0.001
sST2 (ng/ml)	13.7 [8.0-21.6]	10.3 [7.8–16.3]	12.0 [7.1–19.4]	0.25
Gal-3 (ng/ml)	3.4 [2.0-5.9]	3.1 [2.1–5.3]	3.8 [2.0-6.4]	0.87

*N/A = Non-applicable P-value < 0.05 is considered significant

**Fig. 1:**
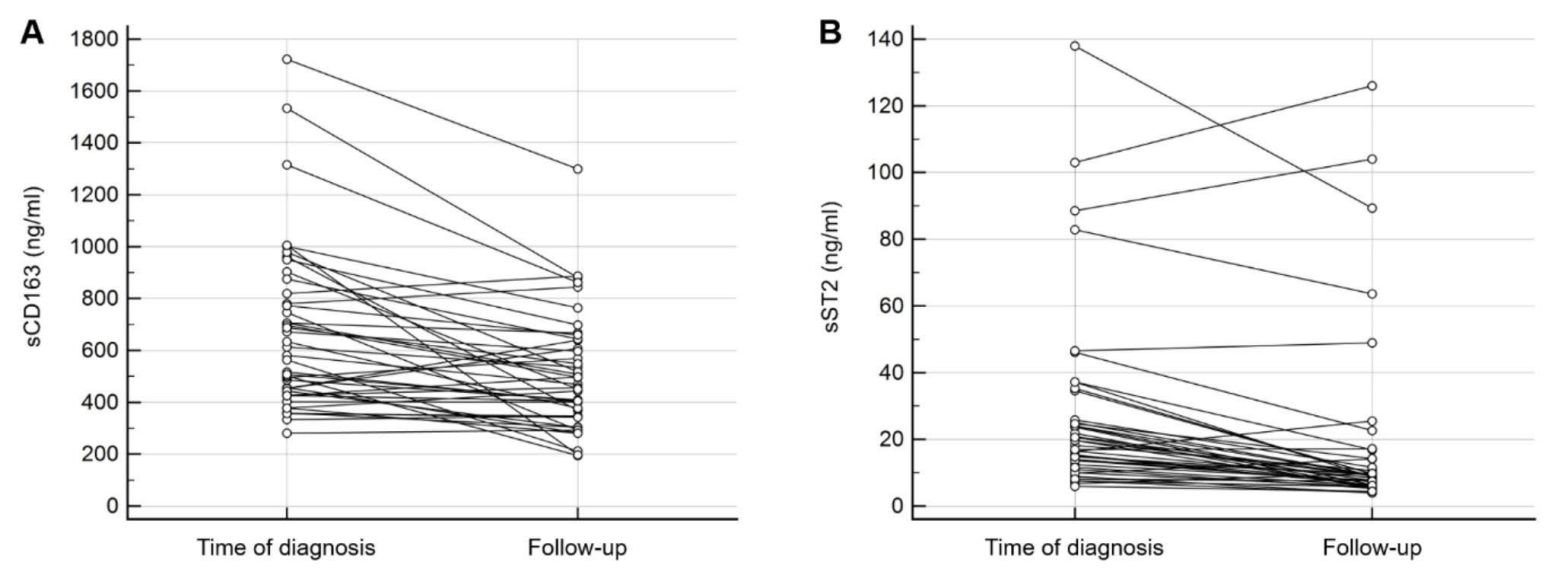
**Circulating sCD163 and sST2 levels in type 1 diabetes patients (n = 43) at time of diagnosis and at follow-up (mean time 1.3 ± 0.5 years).** Plasma levels of both sCD163 and sST2 were elevated at time of diagnosis compared follow-up (p < 0.001 for both)

For patients where blood was drawn at time of and after (mean time 1.3 ± 0.5 years) diagnosis diabetes, the difference in sCD163, sST2 and Gal-3 levels was analysed. Circulating sCD163 was shown to be higher at time of diagnosis compared to after diagnosis (666 ± 318ng/ml and 505 ± 223ng/ml respectively; p < 0.001; Fig. 1 panel A). Plasma levels of sST2 could be observed to be twice as high at onset of type 1 diabetes compared to after diagnosis (18.2 [12.7–25.6] ng/ml and 9.1 [6.3–13.5] ng/ml respectively; p < 0.001; Fig. 1 panel B). Gal-3 levels did not differ from onset of diabetes to after diagnosis (p = 0.29).

In type 1 diabetic patients with a diabetes duration < 5 years, plasma levels of sCD163 were correlated to sST2 (r_s_=0.20, CI^95^: 0.03–0.36; p = 0.018) and Gal-3 (r_s_=0.24, CI^95^: 0.07–0.39; p = 0.005). sST2 and Gal-3 were also observed to correlate in these patients (r_s_=0.33, CI^95^: 0.17–0.47; p < 0.001). HbA1c was shown to correlate to sCD163 (r_s_=0.36, CI^95^: 0.20–0.51; p < 0.001), sST2 (r_s_=0.22, CI^95^: 0.04–0.38; p = 0.016) and Gal-3 (r_s_=0.21, CI^95^: 0.03–0.37; p = 0.020) in patients with a diabetes duration < 5 years.

In patients with a diabetes duration of 5–10 years, plasma levels of sCD163 were correlated to sST2 (r_s_=0.28, CI^95^: 0.05–0.48; p = 0.016) and Gal-3 (r_s_=0.25, CI^95^: 0.01–0.45; p = 0.038). Also in this patient group, sST2 and Gal-3 were shown to correlate (r_s_=0.45, CI^95^: 0.25–0.62; p = 0.001). HbA1c was not correlated to sCD163 (p = 0.48) or Gal-3 (p = 0.17), but to sST2 (r_s_=0.30, CI^95^: 0.07–0.50; p = 0.012) in this patient group.

In the limited number of patients with a diabetes duration of > 10 years included in this study, plasma levels of sCD163 was found not to correlate to sST2 (p = 0.74) or Gal-3 (p = 0.87). A positive correlation could still be observed for sST2 and Gal-3, however (r_s_=0.58, CI^95^: 0.29–0.77; p < 0.001). HbA1c was not correlated to sCD163 (p = 0.26), sST2 (p = 0.73) or Gal-3 (p = 0.95) in this patient group.

## Discussion

In this study, we investigated plasma levels of sCD163, sST2 and Gal-3 at diagnosis of type 1 diabetes in children and adolescents. Circulating sCD163 was higher at the diagnosis of type 1 diabetes and the levels increased with higher HbA1c. This association was lost in patients with a diabetes duration over 5 years. The levels of sCD163 also significantly decreased over time. A previous study has shown that sCD163 is higher in male adults with type 1 diabetes compared to healthy controls and that tumour necrosis factor-like weak (TWEAK), inducer of apoptosis) and ligand to membrane-bound CD163, is associated with complications [14]. In a small study of nine male adults with type 1 diabetes, there was an increase in sCD163 during the early stages of ketoacidosis that was induced by lipopolysaccharide exposure and lack of insulin [26]. In our study, sCD163 was correlated to HbA1c at type 1 diabetes diagnosis. Unfortunately, information regarding ketoacidosis at diagnosis was not available in the current study, but a higher HbA1c could more likely lead to ketoacidosis. Since type 1 diabetes is an autoimmune disease where the immune system is involved [1] and circulating sCD163 has shown to be increased in acute inflammatory disorders [27], this could be one reason for higher levels of sCD163 at diagnosis of type 1 diabetes in our study.

Levels of sCD163 have previously been studied in diabetes-related nephropathy. The Diabetes Incidence Study in Sweden showed a tendency of higher sCD163 in young patients (15–34 years) at the onset of diabetes who later developed nephropathy compared to those who did not [20].

Levels of sST2 were twice as high at the onset of type 1 diabetes compared to after diagnosis and were also correlated to HbA1c in patients with a diabetes duration < 5 years in this study. There are not many studies on sST2 and diabetes complications. We have previously shown that plasma levels of sST2 were significantly higher at the time of onset of diabetes in subjects who, within 10 years of diabetes diagnosis, developed nephropathy [20]. Therefore it would be interesting to continue following children and adolescents also in our ongoing study.

Gal-3 correlated to levels of sCD163 and sST2 in children and adolescents with a diabetes duration < 5 years. Studies on Gal-3 and diabetes have primarily found correlations to metabolic syndrome (at least three of the following conditions: abdominal obesity, hypertension, insulin resistance, serum high-density lipoprotein) [23]. One study within the Diabetes Control and ComplicationsTrial/ Epidemiology of Diabetes Interventions and Complications (DCCT/EDIC) Study investigated biomarkers of renal tubulointerstitial damage and function in type 1 diabetes with and without diabetic kidney disease. They found plasma Gal-3 concentrations significantly higher in patients with kidney disease than in controls with type 1 diabetes but without kidney disease [28].

Children and adolescents that develop type 1 diabetes will have many years of their lives ahead with the disease. There will come periods in their lives, like puberty, when it will be tough to maintain good metabolic control. During puberty there is an increase of hormones that affect insulin sensitivity [29] and for some adolescents that struggle with type 1 diabetes during puberty, it can be difficult to find motivation. Identifying who had the highest risk for complications before they occurred, perhaps with a blood test, could be motivating the patients to take better care of their diabetes.

Identifying risk factors (besides poor metabolic control) before complications from diabetes occur could also help doctors and nurses working with children and adolescents with type 1 diabetes to improve and personalise their treatment. Previous studies have suggested the association of circulating proteins as biomarkers of vascular disease in both adults and children with type 1 diabetes [9, 10, 30]. The current study observed increased sCD163 in patients with recent-onset type 1 diabetes with levels correlating to HbA1c. These findings may suggest poor glycaemic control to be reflected by increased sCD163 and thus a pro-inflammatory milieu that could result in an elevated risk of future vascular disease, but this is only speculative.

One strength of the study is that it includes all children and adolescents with type 1 diabetes from our area in this period of time. Another strength is that blood samples were analysed with commercially available assays with high sensitivity and low variation. A limitation of the study is the short follow-up time.

In conclusion, sCD163 levels increased in patients with recent-onset type 1 diabetes and the levels increased with higher HbA1c. Patients included in this study will be followed annually until the eventual development of diabetic complications, while continuously studying circulating levels of inflammatory proteins such as sCD163.

## Electronic supplementary material

Below is the link to the electronic supplementary material.

Supplementary Material 1
