# Supplementary material for: HbA1c levels and circulating inflammatory proteins at onset of type 1 diabetes in children and adolescents
Source: J Diabetes Metab Disord. 2022 Jul 7;21(2):1427–32. doi: 10.1007/s40200-022-01075-3 (PMC9672210; doi:10.1007/s40200-022-01075-3)
Supplement: Supplementary file 1 — Supplementary Material 1 [file 40200_2022_1075_MOESM1_ESM.docx]

Dear Editor-in-chief

We would appreciate if you considered the enclosed manuscript entitled “Title: HbA1c levels and circulating inflammatory proteins at onset of type 1 diabetes in children and adolescents*”* for submission.

In this study we wanted to investigate plasma levels of sCD163, sST2 and Gal-3 at diagnosis of type 1 diabetes in children and adolescents andfollow the levels of these soluble proteins with diabetes progression as well as studying their correlation to HbA1c.

Kind regards

Charlotta Nilsson

MD, PhD

Department of Pediatrics

Department of Clinical Science

Helsingborg Hospital

Lund University
S- 251 87 Helsingborg

Sweden

E-mail: [Charlotta.Nilsson@med.lu.se](mailto:Charlotta.Nilsson@med.lu.se)
